# A Comprehensive Comparison between Primary Liver Cancer and Liver Metastases through scRNA-Seq Data Analysis

**DOI:** 10.3390/metabo14020090

**Published:** 2024-01-26

**Authors:** Shuang Hao, Liqun Chen, Wenhui Du, Huiyan Sun

**Affiliations:** 1 School of Artificial Intelligence, Jilin University, Changchun 130012, China; haoshuang21@mails.jlu.edu.cn (S.H.); clq23@mails.jlu.edu.cn (L.C.); duwh21@mails.jlu.edu.cn (W.D.); 2 International Center of Future Science, Jilin University, Changchun 130012, China

**Keywords:** metastatic liver cancer, single-cell RNA-Seq, cell proliferation, comprehensive comparison

## Abstract

Metastasis is one of the leading causes of cancer-related deaths. A comprehensive comparison of the differences between primary and metastatic cancers within the same organ can aid in understanding the growth mechanisms of cancer cells at metastatic sites, thereby helping to develop more effective targeted treatment strategies. Primary liver cancer is one of the most common types of cancer, and the liver is also one of the main metastatic sites. In this paper, we utilize single-cell RNA-Seq data to compare primary liver cancer and colorectal liver metastases from multiple perspectives, including cell types and proportions, activity of various cell types, cell–cell communication, mRNA expression differences within the same types of cells, key factors associated with cell proliferation, etc. Our analysis results show the following: (i) Compared to primary tissue, metastatic tissue contains more cytotoxic T cells and exhausted T cells, and it retains some specific characteristics of the primary site. (ii) Cells of the same type exhibit functional differences between primary and metastatic cancers, with metastatic cancer cells showing lower metabolism levels and immune cells exhibiting stronger immune activity. (iii) Interactions between monocytes and hepato-associated cells are strong in primary cancer, while depleted T cells frequently communicate with hepatocytes in metastatic cancer. (iv) Proliferation-related genes in primary and metastatic cancers are mainly involved in cell energy supply and basic metabolism activity, respectively.

## 1. Introduction

Metastatic cancer is cancer that has spread from where it started to another distant organ(s), where cancer cells grow uncontrollably, and this is the primary cause of death for 90% of patients with cancer [1,2]. Despite metastasis being the key cause of the failure of cancer therapy, and of mortality, its relevant mechanisms remain poorly understood. It is obvious that there are fundamental differences in the tumor microenvironment and growth drivers between metastatic cancer and primary cancer [3,4]. Therefore, through comprehensive comparative analysis, we can understand their differences and progression mechanisms at the molecular level, which is conducive to assisting in the treatment of metastatic cancer by halting cancer cell growth [5].

Uncontrolled cell proliferation is one of the most prominent characteristics of cancers. Existing studies have suggested differences between primary and metastatic cancers in the drivers promoting cell proliferation and their mechanisms [6]. Primary cancer grows at the site where the tumor first occurs, while metastatic cancer forms after cancer cells migrate from the primary tumor site to other tissues or organs. This means that metastatic cancer needs to adapt to a new growth environment, including different cell types, matrices, and growth factors [7,8]. Compared to primary cancer, metastatic cancer cells usually have a stronger invasiveness and migration ability. This enables them to penetrate the basement membrane, enter blood vessels or the lymphatic system, and form new tumors at sites distant from the primary tumor [9,10]. Moreover, metastatic cancer cells may be more prone to developing drug resistance than primary cancer cells. This may be due to the selection pressure experienced by metastatic cancer cells in the new growth environment, leading them to develop resistance to treatment [11]. Regarding intratumoral heterogeneity, there are significant differences between primary and metastatic cancers, which means that they have different biological characteristics, affecting the choice and effectiveness of treatment strategies [12,13]. Although existing clinical observations and computational experiments have demonstrated that there are differences in the tumor microenvironment between metastatic and primary cancers, most of them have focused on the differences in a specific protein or molecular component, or compared one tumor at its primary and metastatic sites. For example, Bernardo Cacho-Díaz compared the tumor microenvironment between primary lung and breast cancers and their brain metastases, obtaining results on the establishment of tumor cells in neuronal ecological niches, the upregulation of the expression of several proteins and miRNAs, mutations, and specific epigenetic changes [14]. Mariya Rozenblit et al. conducted a comparison of the PD-L1 protein between primary tumors and metastatic lesions of breast cancer, indicating differences in the immune microenvironment at the metastatic site [15]. In sum, primary and metastatic cancers may be fundamentally driven by different biological and environmental stresses. Primary cancer may be influenced by the local tissue environment, while metastatic cancer needs to cope with challenges in the new growth environment, such as the immune system, hypoxia, and nutrient supply [16,17]. Therefore, we planned to compare primary and metastatic cancers occurring in the liver to clarify the biological differences between the two.

Compared to bulk omics data, single-cell high-throughput sequencing technologies provide a more detailed and nuanced view of biology, allowing researchers to explore the complexity of life at the level of individual cells [18]. This provides a much higher resolution of cellular differences and a better understanding of the function of an individual cell in the context of its microenvironment, allowing for the study of heterogeneity within a cell population and the identification of new cell types in cancer tissues [19,20]. Moreover, single-cell analysis can help us to understand how cells interact with one another, which is important in many biological processes, including development, homeostasis, and disease [21].

In this study, considering that primary liver cancer is one of the most common types of cancer, and that the liver is also one of the main metastatic sites, we collected scRNA-Seq data for four sets of metastatic liver cancer from the colon and four sets of primary liver cancer to comprehensively compare the differences between primary and metastatic cancers. We first compared the differences in cell composition between metastatic cancer and primary cancer, which were determined by single-cell clustering and annotation. On this basis, we conducted a comparative analysis of the communication between various types of cells in metastatic and primary cancers, especially between liver cells and various immune cells, which is helpful to reveal the mechanisms of tumor immunosuppression and immune escape, and to assist with targeted immunotherapy. Furthermore, at a fine-grained level, we analyzed the gene expression differences of the same cell types in primary and metastatic cancer tissues to explore their substantial changes in cell state in different microenvironments. Considering batch effects among different datasets, we developed a rank-based differential expression analysis method to make the gene expression of different samples comparable. Furthermore, uncontrolled cell proliferation is an important characteristic of cancer for both primary and metastatic cancers, but, due to differences in the microenvironment, we suspect that the driving forces of cell proliferation are different. Hence, we separately selected and then compared the genes that were highly correlated with the cell proliferation level in each group to infer the drivers promoting cell proliferation. Overall, we made an extensive comparison between metastatic and primary cancers from multiple scales and perspectives and analyzed their differences, which helped us to better understand the mechanism of cell proliferation of metastatic cancer and provided new insights into more effective treatments for both primary and metastatic cancers.

## 2. Materials and Methods

### 2.1. Overview

**Figure 1:**
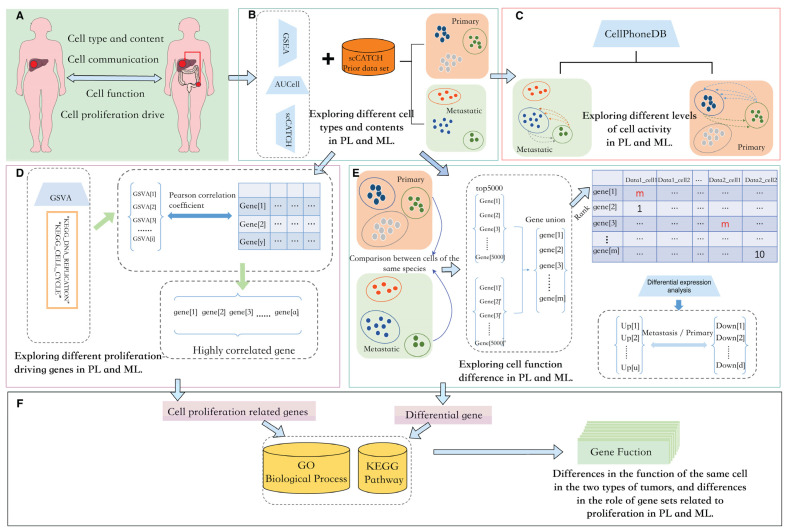
Overview of our study. (**A**): We mainly carry out four studies on metastatic liver cancer and primary liver cancer: cell type and content, cell communication, cell function, and cell proliferation. (**B**): Cell annotation is completed using GSEA, AUCell, and scCATCH paired with a priori gene sets included in scCatch. (**C**): CellPhoneDB is used to calculate the communication strength of various types of cells. (**D**): GSVA calculates the enrichment of genes in the tissue in proliferation-related pathways and then we select genes with high Pearson correlation coefficients. (**E**): Differential analysis of similar cells in two tumor types uses a rank-based approach. (**F**): The proliferation-related genes and cell differential genes selected in Figures (**D**,**E**) are enriched in the GO:BP and KEGG databases. PL: primary liver cancer. ML: metastatic liver cancer.

In this study, we planned to investigate primary and metastatic liver cancer from four aspects: cellular composition, cellular communication, cellular function, and cellular proliferation drive, as shown in Figure 1A. We hoped to obtain the differences in cell composition and content between primary and metastatic cancers through the integrated analysis of cell annotation tools such as Gene Set Enrichment Analysis(GSEA) [22], the activity of these regulons is quantified via an enrichment score for the regulon’s target genes( AUCell) [23], and single cell Cluster-based Annotation Toolkit for Cellular Heterogeneity(scCATCH) [24], as shown in Figure 1B. Following this, we planned to use CellPhoneDB to distribute the communication of each part of the cells as in Figure 1C. We also paid attention to cell proliferation genes in tumor tissues. Gene set variation analysis (GSVA) was used to calculate the enrichment scores of each cell in the “KEGG_DNA_REPLICATION” and “KEGG_CELL_CYCLE” pathways, and Pearson correlation coefficients were used to select the highly relevant genes as shown in Figure 1D. In the two types of tumors, although the same type of cells are bound to have differences in specific functions, we planned to obtain differential genes through rank-based differential analysis., as in Figure 1E. Analysis based on a single gene made it difficult to reflect biological characteristics. To explore the function of feature genes, we conducted Gene Ontology Biological Process(GO:BP) [25] and Kyoto Encyclopedia of Genes and Genomes(KEGG) [26] enrichment analysis for proliferation-related genes and differential genes of various parts to obtain the specific functions of these feature genes, as shown in Figure 1F. The specific processes and tools are shown in Figure 1.

### 2.2. Data Collection and Processing

We collected scRNA-Seq data for four sets of metastatic liver cancer from the colon and four sets of primary liver cancer from the Gene Expression Omnibus (GEO) [27] database, respectively. This provides a total of 44 samples and 133,129 cells (as shown in Table 1).

To obtain high-quality gene expression data for downstream analyses, we first removed the following genes and cells: (i) genes that express in less than 3 cells and (ii) cells that have less than 200 genes. According to the distribution of each group of datasets, cells containing an excess of mitochondrial genomes, ribosomal genomes, or red blood cell genes were excluded. For datasets containing multiple GEO Samples(GSM), we used the harmony [28] algorithm to avoid the batch effect by correcting and integrating multiple sets of data.

In addition, “NormalizeData” was employed for normalizing the data to make expression of the same gene comparable among different samples. “FindVariableFeatures” in Seurat (v4) [29] was used to identify highly variable genes, and then the “ScaleData” function was applied to standardize the data.

### 2.3. Dimension Reduction and Cell Type Annotation

Based on normalized and standardized data, we further used principal component analysis (PCA) [30] on the top 2000 highly variable genes to reduce the dimensions for better clustering and visualization. The ‘FindNeighbors’ and ‘FindClusters’ functions were used for automatic clustering analysis.

To determine the cell type of each cluster more accurately and robustly, we proposed to perform an ensemble analysis by simultaneously using the GSEA, scCATCH, and AUCell methods. GSEA and scCATCH were used as the main methods for predicting cell types. Due to that the basic principles of these two cell annotation approaches being different, their cell annotation results may inevitably be inconsistent. For the cell cluster with conflicting results, AUCell was utilized to calculate the activity level of the signature gene set in the two cell types, and the cell type with the higher activity of the signature gene set was taken as the final annotation result of the cluster.

ScCATCH contains 20,792 marker genes from 2097 references, including 184 tissue types, 353 cell types, and 686 cell subtypes. In our study, we employed 49 cell types from human liver and colorectal cancer tissues in scCATCH as prior information for cell annotation. The “findcelltype” function within the scCATCH package was used to assign cell types to each cell cluster. In GSEA analysis, the “fgsea” function in the fgsea package was utilized to perform bioprocess enrichment analysis on the set of marker genes in each cell cluster relative to the individual cell type in the a priori set of genes. The cell type with the highest NES (normalized enrichment score) was selected as the annotation result for the cell cluster. When using AUCell for final annotation decision row selection, the “AUCell_buildRankings” function in the AUCell package was first used to rank gene expression, and then the “AUCell_calcAUC” function was applied to score the cells based on the a priori gene sets of genes to determine the cell type.

### 2.4. Rank-Based Differential Expression Analysis

To capture the molecular difference between primary and metastatic cancers at the cell level, we undertook hypothesis testing analysis to identify the significantly differential gene expression for each cell type. Due to the data used in this study coming from different laboratories, the batch effect was a challenging problem.

Although some existing methods are able to eliminate the batch effects of single-cell data, they inevitably eliminate some real differences in biology or mistake batch effects as biological differences. Therefore, traditional hypothesis-testing methods based on gene expression distribution are not applicable. In response to this situation, Wang H et al. proposed the RankComp method [31], which assumed the rank of gene expression in each sample was stable and converted the expression value of each gene into ranking in each sample and identified gene pairs with relatively stable expression in the data. Inspired by this idea, we also used the rank of gene expression instead of expression values and used the degree of change in rankings as a criterion to test the differences.

We first calculated the top 5000 genes with average expression values in the same cell type for each dataset. For each cell type, we merged the expression of each dataset into an *m* × *n* matrix, where m represents the union length of the expressed genes in each set, and *n* represents the number of cells after merging. In each column of data, that is, in each cell, we sorted the gene expression values in descending order and ranked the highest expression value as 1. As single-cell data are usually sparse with a large number of 0 expression values, in the rank matrix, we unified the rank of genes whose expression value was 0 to m. We performed a fold-change and *t*-test analysis on the rank matrix between the metastasis cancer group and the primary cancer group. The formulas are as follows:

(1) $$FC=\frac{mean\left(X_{i}\right)}{mean\left(Y_{i}\right)}$$

(2) $$t=\frac{mean\left(X_{i}\right)-mean\left(Y_{i}\right)}{s_{i}\sqrt{\left(\frac{1}{n_{x}}+\frac{1}{n_{y}}\right)}}$$

where $mean\left(X_{i}\right)$ and $mean\left(Y_{i}\right)$ are the average rank of gene *i* in the metastatic cancer data and primary cancer data, respectively. And $s_{i}$ is the standard deviation of gene *i* across all samples, $n_{x}$ and $n_{y}$ are the number of cells of metastatic cancer and primary cancer, respectively.

### 2.5. Enrichment Analysis

To explore the functions of differentially expressed genes, the “Clusterprofiler” [32] package was used for both gene ontology (GO) and the Kyoto Encyclopedia of Genes and Genomes (KEGG) enrichment analysis of upregulated and downregulated genes, respectively. We chose appropriate significance thresholds and presented the results by the ggplot2 package [33].

### 2.6. Cell Crosstalk Analysis

We performed a comparative analysis of the communication activity between cells in primary and metastatic cancers. CellPhoneDB [34] is a database containing receptor–ligand pairs, which facilitates comprehensive and systematical analyses of communication between cells, and the construction of communication networks between different cell types. We used the “statistical_analysis” method in CellPhoneDB for analysis.

### 2.7. GSVA Enrichment and Correlation Calculation

To quantify the proliferation level of each cell and then compare the proliferation level difference between metastatic cancer and primary cancer, we first calculated the enrichment score of tumor-associated cells on the “KEGG_DNA_REPLICATION” and “KEGG_CELL_CYCLE” pathways, which strongly represent the proliferative capacity of cells, by using the “gsva” function in the GSVA [35] package. Then, we inferred the driver promoting cell proliferation by calculating the Pearson correlation coefficient between individual genes and the proliferation enrichment scores for each cell type and selected genes with both *p*-values less than 0.05 and correlation coefficients greater than 0.3 for subsequent analysis.

## 3. Results 

### 3.1. Comparison of Cell Components between Primary and Metastatic Liver Carcinoma

**Figure 2:**
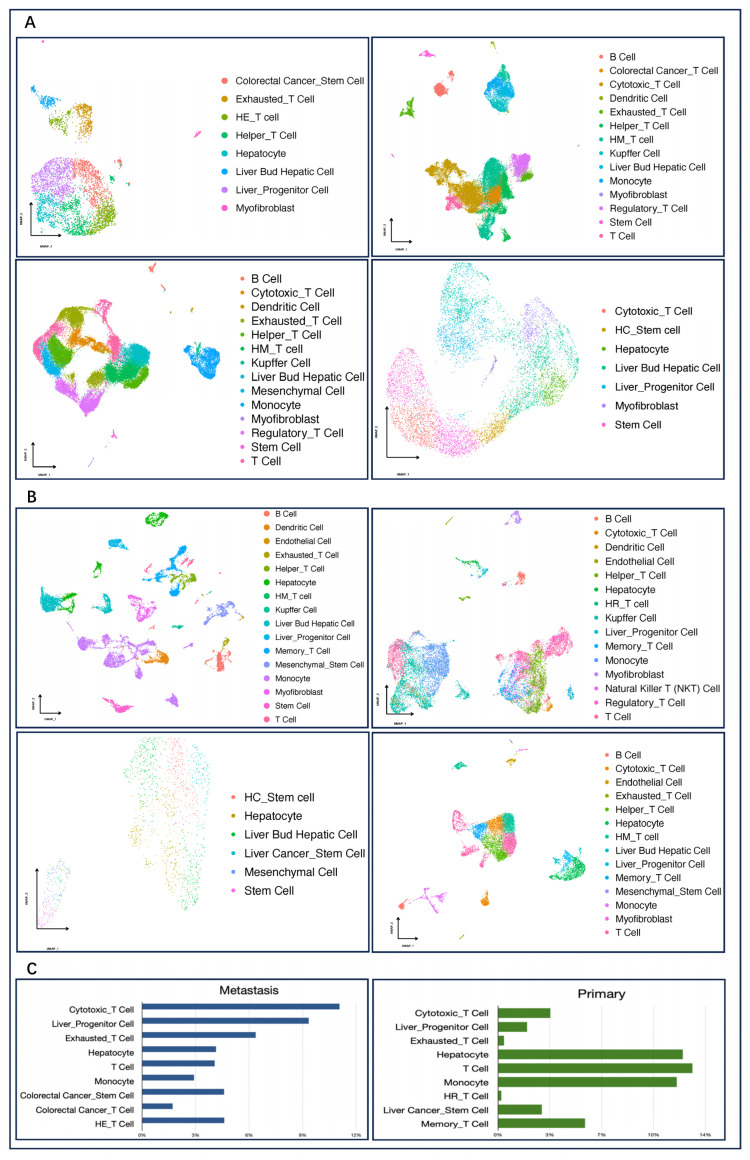
Cell composition of primary and metastatic cancers. (**A**): Cell annotated images of four sets of colorectal cancer liver metastasis data, in order: GSE158692, GSE164522, GSE178318, GSE225857; (**B**): Annotated images of cell types for four sets of primary liver cancer data, in order: GSE149614, GSE166635, GSE188289, GSE210679. (**C**): Comparison of the percentage content of each cell type in primary and metastatic cancers. HE_T cell:Hepatocellular Cancer_Exhausted_T Cell; HM_T cell: Hepatocellular Cancer_Mucosal-Associated Invariant_T Cell; HC_Stem cell:Hepatocellular Cancer_Stem Cell; HR_T cell: Hepatocellular Cancer_Regulatory_T Cell.

Through conducting a systematic analysis of metastatic liver cancer and primary liver cancer tissues, we predicted individual cell types by unsupervised clustering which was performed with Seurat v4. Through integrating GSEA, scCATCH, and AUCell with the cell-type marker genes in the scCATCH database to annotate these cell clusters, 27 cell types were identified across these eight sets of data as shown in Figure 2A,B. It was found that the metastatic liver tissues mainly included 20 types of cells such as cytotoxic T cells, liver progenitor cells, exhausted T cells, colorectal cancer stem cells, and mucosal-associated invariant T (MAIT) cells. On the other hand, primary liver cancer tissues mainly included 23 types of cells such as monocytes, T cells, hepatocytes, and Kupffer cells, among others.

To further analyze the content difference of various cells in the two types of liver cancer tissue, we calculated and compared the proportion of each cell type, as shown in Figure 2C. Compared with primary liver cancer, the content of cytotoxic T cells, liver progenitor cells, and exhausted T cells in metastatic liver tissue of colorectal cancer is significantly higher but significantly lower in hepatocytes, T cells, and monocytes. Since the metastatic cancer cells here are derived from colorectal tissue, colorectal cancer stem cells and immune cells with colorectal characteristics are present in the metastatic tissues. In primary liver cancer, the cancer cells come from the liver tissue themselves, and liver characteristics in it are more obvious. Cytotoxic T cells, as an important subtype of T cells, are the main force against cancer cells. Exhausted T cells originate from a special differentiation state that cytotoxic T cells enter after long-term exposure to antigens, and they are also one of the main obstacles to anti-tumor immunity during tumor development [36].

Based on the content difference, we speculated that in response to emerging metastases, the immune system mobilizes more cytotoxic T cells in an attempt to destroy the cancer cells. However, it fails to completely inhibit cancer cell proliferation, and a large number of T cells that are involved in tumor immune activity but fail to eliminate cancerous tissues enter a state of exhaustion. There is growing evidence that exhausted T cells can undergo metabolic dysfunction, accompanied by alterations in the signaling cascade and epigenetic background that inhibit immunity [37] and lead to a vicious cycle.

### 3.2. Different Functions of the Same Cell Type in Primary and Metastatic Liver Cancer

**Figure 3:**
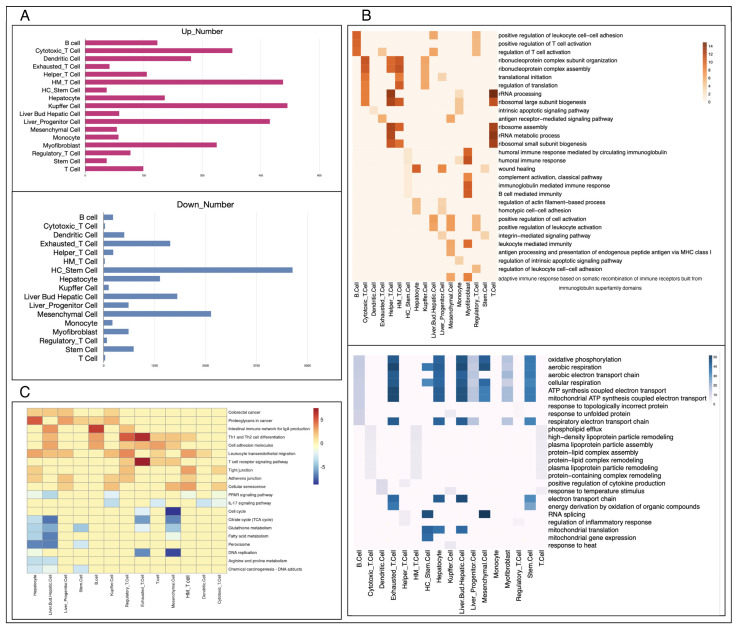
Functional difference analysis of the same cell type in two types of tumors. (**A**): The number of up- and downregulated genes in each cell type, the figure above is downregulated and the one below is upregulated. (**B**): The GO BP enrichment results of differential genes between primary and metastatic cancers, the figure above is upregulated and the one below is downregulated. (**C**): The results of KEGG enrichment of differentiated genes in primary and metastatic cancers. HE_T cell:Hepatocellular Cancer_Exhausted_T Cell; HM_T cell: Hepatocellular Cancer_Mucosal-Associated Invariant_T Cell; HC_Stem cell:Hepatocellular Cancer_Stem Cell; HR_T cell: Hepatocellular Cancer_Regulatory_T Cell.

Due to the different growth drivers and microenvironments between primary and metastatic cancers, in addition to comparing cell types and proportions in tissues, we further analyzed the significant differences at the molecular level between cells of the same type in primary and metastatic cancers. According to the annotation results, a total of 17 cell types, including B cells, exhausted T cells, hepatocytes, and others, are common to both metastatic and primary cancers. We conducted rank-based differential analyses on these 17 cell types as shown in Figure 3A. Hepatocellular cancer stem cells, a subset of hepatocellular carcinoma cells with stem cell properties, drive the growth of primary liver cancer due to their unique stem-cell-like self-renewal and differentiation ability [38]. Mucosal-associated invariant T (MAIT) cells are innate-like T cells and have been demonstrated to promote tumor initiation, growth, and metastasis by inhibiting T and/or NK cells [39]. 

To explore the functions of these differentially expressed genes, we performed KEGG and GO biological processes enrichment analyses on genes of each cell type (Figure 3B,C) and detailed results of enrichment analysis were shown in Supplementary materials Tables S1 and S2. Through KEGG pathway enrichment analysis, we observed the upregulated genes in hepatocytes within metastatic liver cancer were associated with tight junctions (*p*-value = 1.47 × 10^−3^) and proteoglycans (*p*-value = 4.72 × 10^−8^), while the downregulated genes in metastatic cancer were mainly enriched in various metabolic pathways, including glutathione metabolism (*p*-value = 4.29 × 10^−7^) and fatty acid metabolism (*p*-value = 1.73 × 10^−8^) in liver bud hepatic cells, arginine and proline metabolism (*p*-value = 4.19 × 10^−5^) as well as the citrate cycle (*p*-value = 5.75 × 10^−5^) in hepatocytes. In addition, we observed that metastatic cancers retain some characteristics of their primary location. For example, in colorectal cancer liver metastases, some genes in the key pathways related to colorectal cancer are significantly expressed in hepatocytes (*p*-value = 8.10 × 10^−4^) and liver progenitor cells (*p*-value = 3.60 × 10^−4^).

The GO biological process (BP) enrichment results showed that compared with primary cancer, upregulated genes in metastatic cancer mainly regulated the activity of immune cells such as B cells (*p*-value = 1.63 × 10^−10^), as well as the homotypic cell adhesion (*p*-value = 1.57 × 10^−5^) and wound-healing processes (*p*-value = 3.69 × 10^−11^) of hepatocytes. Similarly, the results of GO enrichment showed that the downregulated genes of metastatic cancer were mainly enriched in metabolic activities such as ATP synthesis (*p*-value = 3.89 × 10^−49^) and oxidative phosphorylation (*p*-value = 3.07 × 10^−45^) in exhausted T cells and aerobic electron transport chains (*p*-value = 2.29 × 10^−43^) in liver bud hepatic cells. Compared with normal tissues, malignant tumors are loose inside, and some cells can easily escape and penetrate the blood circulation to metastasize to other organs or even throughout the body [40]. In our analyses, we observed proteins that respond to cell adhesion, such as tight junctions and homotypic cell adhesion, were significantly upregulated in metastatic cancer. Combined with this result, we speculate that the aggregation of cancer cells free in the bloodstream to form distal solid tumors requires stronger cell adhesion than the primary cancer tissue possesses. Compared to primary cancer, the microenvironment of metastatic cancer cells changes greatly, which leads to metabolic reprogramming. On the other hand, it is important for metastatic cancer cells to escape the supervision of the immune system, achieve coordination with the new environment by adjusting its metabolic state, and finally achieve remote colonization [41]. 

### 3.3. Diverse Cell–Cell Communications in Primary and Metastatic Liver Cancer

**Figure 4:**
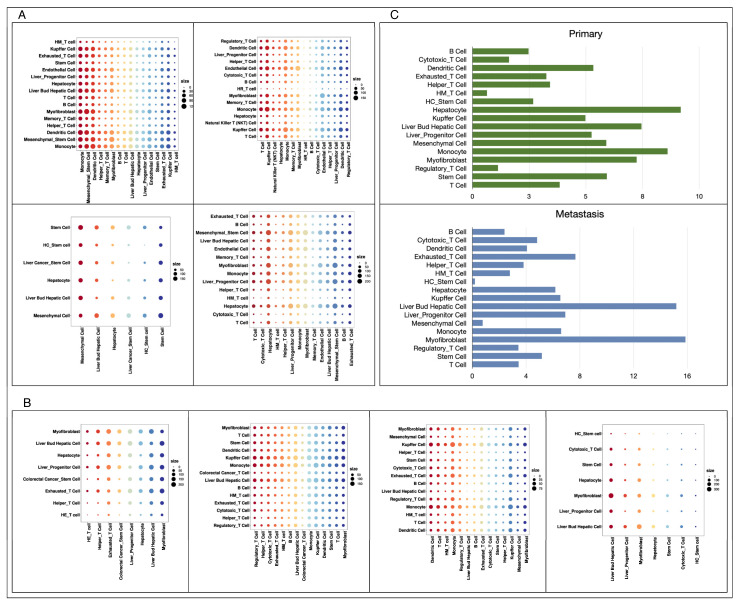
Cell communication intensity. (**A**): Chords diagram of cell interaction intensity for four primary liver tumors, in order: GSE149614, GSE166635, GSE188289, GSE210679. (**B**): Chords diagram of cell interaction intensity for four groups of colorectal cancer liver metastasis data, in order: GSE158692, GSE164522, GSE178318, GSE225857. (**C**): Comparison of the communication intensity of the same cell types in primary and metastatic cancers, with the horizontal coordinate representing the percentage of the number of communication paths for the current cell type in the total number of communication paths in the data.

We analyzed cell communication in metastatic and primary cancer tissues separately by using CellPhoneDB, which measures the activity degree of cell-type-specific receptor and ligands in each set of data, and the results are shown in Figure 4A and Figure 4B, respectively. The experimental results showed that there was no significant difference in the overall number of receptors and ligands between metastatic cancer and primary cancer tissue cells, but the number of receptor–ligand pairs contained in each cell type varied considerably, as illustrated in Figure 4C. In primary liver cancer tissues, hepatocytes are the cell type containing the most receptor–ligand pairs, followed by monocytes and liver progenitor cells, while in metastatic liver cancer originating from colorectal cancer, myofibroblasts become the members containing the most receptor–ligand pairs, followed by liver bud hepatic cells and exhausted T cells.

We observed that communication between monocytes and liver-associated cells was more pronounced in primary hepatocellular carcinoma tissues, which was consistent with previous studies that hepatocellular carcinoma cells were able to deliver signaling molecules to monocytes in the form of microvesicles, and this significantly enhanced the activity of monocytes and accelerated their differentiation [42]. According to subsequent analysis, we found that a greater proportion of T cells in the metastatic tissues were exhausted, the effector functions of exhausted T cells were lost, and the transcriptional profiles as well as the metabolic profile shifted [43], which may promote intensive interaction between exhausted T cells and liver bud hepatic cells. We also observed a large amount of crosstalk between myofibroblasts and hepato-associated cells in primary cancer, which is consistent with previous studies reporting that hepatocellular carcinoma is closely related to liver fibrosis and myofibroblasts are an important component of liver fibrosis [44].

### 3.4. Biological Processes Related to Cell Proliferation in Primary and Metastatic Liver Cancer

In primary tumors, cancer cells proliferate at their own location, whereas metastatic cancer cells originate from one site and colonize a new location by invading lymph vessels and blood vessels and undergoing a series of complex processes. The different sources and microenvironments of cancer cells may indicate different proliferation abilities and driving forces. Clearly understanding the proliferation mechanisms of different cancer cells contributes to exploring more effective interventions and treatments.

To answer this question, we used GSVA to calculate the enrichment scores of two cell-proliferation-related pathways, DNA replication, and the cell cycle in liver-associated cells. To obtain the driver elements that may regulate cell proliferation in individual cells, we first calculated the Pearson correlation of each gene with these two pathways in tumor cells in metastasis and primary cancer, respectively. And then we separately selected the genes with both *p*-values less than 0.05 and correlation coefficients greater than 0.3, and the number of related genes in each dataset is shown in Table 2.

**Figure 5:**
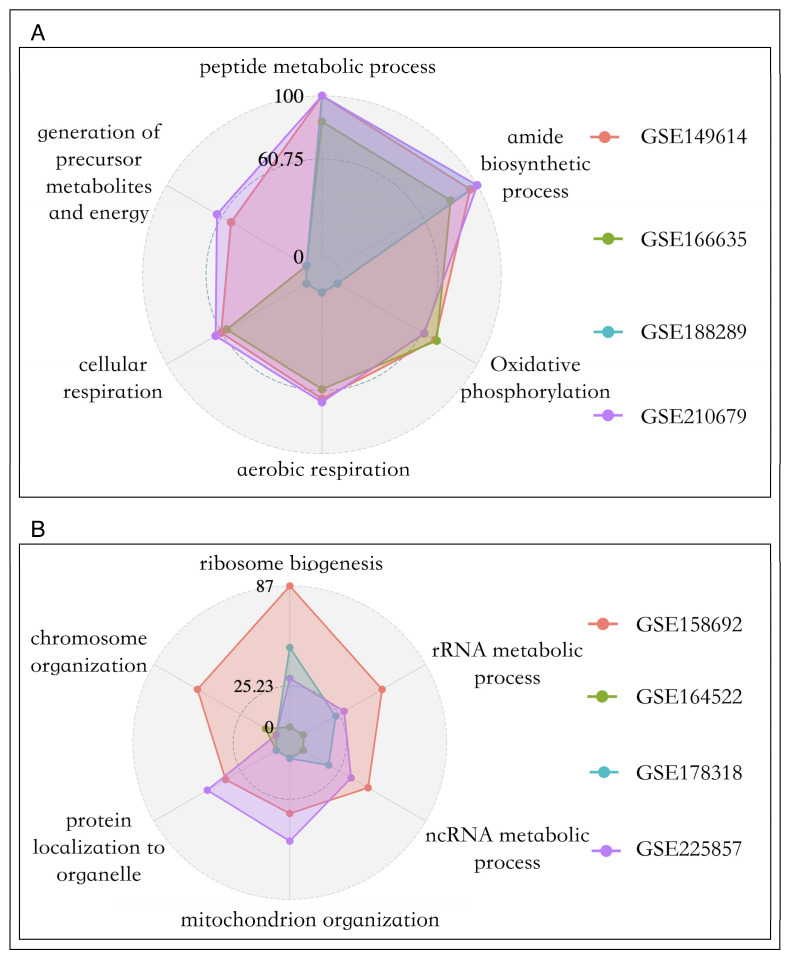
Pathway enrichment bar chart of cell proliferation driven genes. (**A**): Primary cancer. (**B**): Metastatic cancer.

We found that across four sets of primary cancer data, there were no genes significantly negatively correlated with the two proliferation pathways. The same phenomenon has been observed in most metastatic cancer datasets, whicI notice that the GSE in Figure 5 is italicized, please confirm that you are invited to change it to italicized. Please note that this should be consistent throughout the texth is in line with the reality of the infinite proliferative properties of cancer cells [45]. Therefore, we conducted functional enrichment analysis for genes positively related to cell proliferation in the two tumors, and the results are shown in Figure 5A and 5B. In primary liver cancer, the genes highly associated with cell proliferation mainly participate in biological processes such as amide biosynthetic process (*p*-value = 1 ×10^−94^), oxidative phosphorylation (*p*-value = 1.78 × 10^−51^), and aerobic respiration (*p*-value = 3.16 × 10^−49^), while genes in metastatic cancer mainly regulate rRNA metabolic processes (*p*-value = 1 × 10^−27^) and ribosome biogenesis (*p*-value = 3.16 × 10^−42^). 

Overall, we found that, unlike the proliferation genes of primary cancer cells, which mainly supply energy to cells, the proliferation genes of metastatic cancer cells were significantly involved in promoting the synthesis of metastatic substances, which was consistent with previous studies. For epithelial tumors, tumor cells need to undergo epithelial-to-mesenchymal transition (EMT) to gain metastatic capability. It has been found that synthesizing new ribosomes can drive EMT, as it helps to synthesize the proteins needed for cellular functions, while inhibiting ribosome biogenesis can prevent EMT [46].

## 4. Discussion

The mortality rate of metastatic cancer remains high mainly due to the lack of a comprehensive understanding of metastatic cancer. As primary liver cancer is one of the most common types of cancer and the liver is also one of the main metastatic sites, in this study, we focused on the comparison of metastatic and primary liver cancer from different scales and perspectives.

Regarding cell composition, we observed that compared to primary liver cancer, the liver metastasis tissue of colorectal cancer not only retained some characteristics of colorectal cancer stem cells but also displayed differences in the types and contents of immune cells. Through analyzing the expression level of specific genes, we observed that the activity of immune cells was higher in metastatic cancer. As tumors progress to the metastatic stage, the interplay and competition between cancer cells and immune cells become increasingly complex, and ultimately makes the immune system powerless against cancer cells [36].

From the perspective of differential gene expression, our analysis results suggested that metastatic cancer cells were less active in some metabolic pathways. In the process of distal organ colonization by cancer cells, the coordination of cancer cell metabolism and organ environment contributes to their colonization [47]. Therefore, tumor cells may independently choose the most suitable energy supply for their growth based on the concentration and content of nutrients in the microenvironment in which they live, affecting the ability to synthesize fatty acids, glutathione, and other organisms [48]. The adaptability of metastatic cancer cells in different environments may depend on the origin of the tumor and the site of metastasis, and further research on the influence of both is very valuable.

Cell proliferation is an important hallmark of both primary and metastatic cancer, and one of the key strategies for cancer therapy is to inhibit the uncontrolled proliferation of tumor cells. Hence it is important to identify the key elements that drive cell proliferation for understanding the cancer mechanism and determining drug targets. Through conducting quantification of cell proliferation level and correlation analysis in primary cancer cells and metastatic cancer cells, we observed that the genes associated with cell proliferation in metastatic cancer mainly participated in various metabolism reactions of RNA, while producing energy in primary cancer. This was highly consistent with our assumptions that the driving forces of cell proliferation in primary cancer and metastatic cancer were different. In further studies, causal inference or molecular biology experiments are needed for validation. In addition, in future work, we will also collect more data for analysis, such as single-cell RNA-seq data on liver metastases of both bowel and other primary organs, to obtain more robust results. As more sequencing data are available, we can also analyze data from other metastases to construct the landscape of metastatic cancer.

In summary, using single-cell transcriptome data, our study described the differences between primary and metastatic liver cancer under multi-scale and multi-level approaches and analyzed the differences in cell proliferation drivers and their respective functions; we believe this was helpful for better understanding tumor mechanisms, as well as the effects of receiving specific cancer therapy.

## Figures and Tables

**Table 1:** Summary of scRNA-Seq data for metastatic and primary liver cancer.

	GSE	Number of Samples	Number of Cells
Metastatic	GSE178318	6	40,170
	GSE225857	2	8482
	GSE158692	6	4587
	GSE164522	17	34,995
Primary	GSE149614	9	20,762
	GSE166635	2	13,696
	GSE188289	1	1410
	GSE210679	1	9027
Total	8	44	133,129

**Table 2:** Summary of proliferation-related genes for metastatic and primary liver cancer.

	GSE	Number of Genes
Metastasis	GSE178318	121
	GSE225857	2849
	GSE158692	1642
	GSE164522	21
Primary	GSE149614	1909
	GSE166635	1699
	GSE188289	1641
	GSE210679	2911

## Data Availability

The datasets used and analyzed during the current study are available from the GEO database at https://www.ncbi.nlm.nih.gov/geo/ accessed on 10 July 2023 (Accession GSE210679, GSE149614, GSE188289, GSE158692, GSE164522, GSE178318, GSE225857).

## Supplementary Materials

The following supporting information can be downloaded at: https://www.mdpi.com/article/10.3390/metabo14020090/s1. Table S1: Comprehensive analysis of differentially expressed genes by KEGG. Table S2: Comprehensive analysis of differentially expressed genes by GO:BP.
